# Bis(2,3-diamino­pyridinium) bis­(μ-pyridine-2,6-dicarboxyl­ato)-κ^4^ *O* ^2^,*N*,*O* ^6^:*O* ^6^;κ^4^ *O* ^2^:*O* ^2^,*N*,*O* ^6^-bis­[aqua­(pyridine-2,6-dicarboxyl­ato-κ^3^ *O* ^2^,*N*,*O* ^6^)bis­muthate(III)] tetra­hydrate

**DOI:** 10.1107/S1600536811005629

**Published:** 2011-02-23

**Authors:** Hossein Aghabozorg, Shokoofeh Kazemi, Ali Akbar Agah, Masoud Mirzaei, Behrouz Notash

**Affiliations:** a Faculty of Chemistry, Islamic Azad University, North Tehran Branch, Tehran, Iran; b Faculty of Chemistry, Tarbiat Moallem University, 15614 Tehran, Iran; c Department of Chemistry, School of Sciences, Ferdowsi University of Mashhad, Mashhad 917791436, Iran; d Department of Chemistry, Shahid Beheshti University, G. C., Evin, Tehran 1983963113, Iran

## Abstract

In the centrosymmetric dinuclear complex anion of the title compound, (C_5_H_8_N_3_)_2_[Bi_2_(C_7_H_3_NO_4_)_4_(H_2_O)_2_]·4H_2_O, the Bi^III^ atom is eight-coordinated in an N_2_O_6_ environment and has a distorted bicapped trigonal–prismatic coordination environment. Extensive inter­molecular O—H⋯O, N—H⋯O and weak C—H⋯O hydrogen bonds lead to the stability of the crystal structure. Inter­actions between one C—H group of the 2,3-diamino­pyridinium [(2,3-dapyH)^+^] cation and the aromatic ring of the pyridine-2,6-dicarboxyl­ate (pydc) ligand (C—H⋯centroid distance = 2.78 Å) and π–π inter­actions between the (2,3-dapyH)^+^ cations and between the (2,3-dapyH)^+^ cation and the pydc ligand [centroid–centroid distances = 3.489 (5) and 3.694 (5) Å] are observed.

## Related literature

For related structures, see: Aghabozorg *et al.* (2008 ▶, 2010 ▶); Sheshmani *et al.* (2005 ▶).
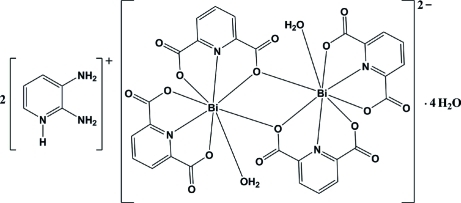


## Experimental

### 

#### Crystal data

(C_5_H_8_N_3_)_2_[Bi_2_(C_7_H_3_NO_4_)_4_(H_2_O)_2_]·4H_2_O
*M* *_r_* = 1406.76
Triclinic, 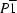

*a* = 9.3462 (19) Å
*b* = 10.726 (2) Å
*c* = 11.098 (2) Å
α = 95.13 (3)°
β = 91.38 (3)°
γ = 90.47 (3)°
*V* = 1107.7 (4) Å^3^
*Z* = 1
Mo *K*α radiation
μ = 8.03 mm^−1^
*T* = 298 K
0.33 × 0.27 × 0.23 mm

#### Data collection

Stoe IPDS-2 diffractometer
Absorption correction: numerical (*X-SHAPE* and *X-RED32*; Stoe & Cie, 2005 ▶) *T*_min_ = 0.083, *T*_max_ = 0.156
12406 measured reflections
5940 independent reflections
5539 reflections with *I* > 2σ(*I*)
*R*_int_ = 0.113

#### Refinement

*R*[*F* ^2^ > 2σ(*F* ^2^)] = 0.057
*wR*(*F* ^2^) = 0.154
*S* = 1.05
5940 reflections
347 parameters
9 restraints
H atoms treated by a mixture of independent and constrained refinement
Δρ_max_ = 2.98 e Å^−3^
Δρ_min_ = −2.93 e Å^−3^

### 

Data collection: *X-AREA* (Stoe & Cie, 2005 ▶); cell refinement: *X-AREA*; data reduction: *X-AREA*; program(s) used to solve structure: *SHELXS97* (Sheldrick, 2008 ▶); program(s) used to refine structure: *SHELXL97* (Sheldrick, 2008 ▶); molecular graphics: *ORTEP-3* (Farrugia, 1997 ▶); software used to prepare material for publication: *WinGX* (Farrugia, 1999 ▶).

## Supplementary Material

Crystal structure: contains datablocks I, global. DOI: 10.1107/S1600536811005629/hy2406sup1.cif

Structure factors: contains datablocks I. DOI: 10.1107/S1600536811005629/hy2406Isup2.hkl

Additional supplementary materials:  crystallographic information; 3D view; checkCIF report

## Figures and Tables

**Table 1:** Hydrogen-bond geometry (Å, °)

*D*—H⋯*A*	*D*—H	H⋯*A*	*D*⋯*A*	*D*—H⋯*A*
N3—H3*A*⋯O11^i^	0.87 (14)	2.02 (14)	2.797 (10)	148 (12)
N4—H4*A*⋯O2^ii^	0.86	2.11	2.924 (9)	158
N4—H4*B*⋯O6^i^	0.86	1.97	2.830 (10)	178
N5—H5*A*⋯O3^iii^	0.86	2.53	3.216 (10)	138
N5—H5*B*⋯O6^i^	0.86	2.11	2.972 (10)	176
O9—H9*A*⋯O8^iv^	0.81 (7)	2.07 (12)	2.746 (10)	141 (15)
O9—H9*B*⋯O11^v^	0.84 (8)	1.99 (10)	2.771 (11)	155 (15)
O10—H10*A*⋯O5	0.94 (8)	1.96 (8)	2.862 (9)	160 (11)
O10—H10*B*⋯O7^vi^	0.80 (8)	2.21 (8)	2.972 (10)	160 (13)
O11—H11*A*⋯O4^iii^	0.89 (8)	2.01 (13)	2.717 (11)	136 (14)
O11—H11*B*⋯O10^vii^	0.92 (9)	1.96 (9)	2.836 (12)	158 (13)
C11—H11⋯O8^viii^	0.93	2.26	3.050 (10)	142

Symmetry codes: (i) 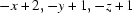; (ii) 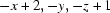; (iii) 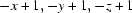; (iv) 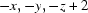; (v) 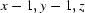; (vi) 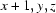; (vii) 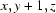; (viii) 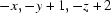.

**Fig. 1.**
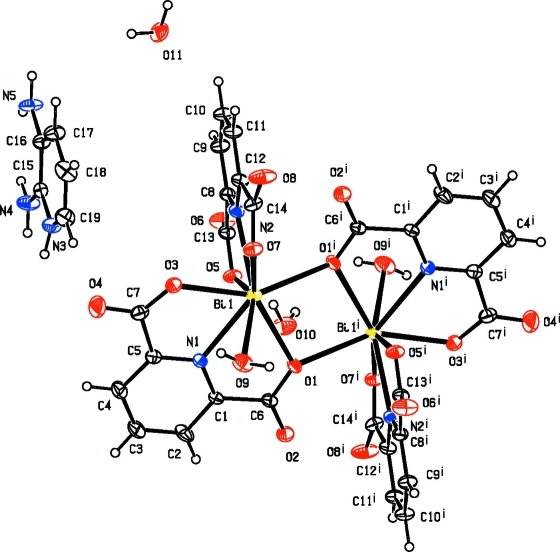
The molecular structure of the title compound, with displacement ellipsoids drawn at the 30% probability level. [Symmetry code: (i) -x+1, -y, -z+2.]

**Fig. 2.**
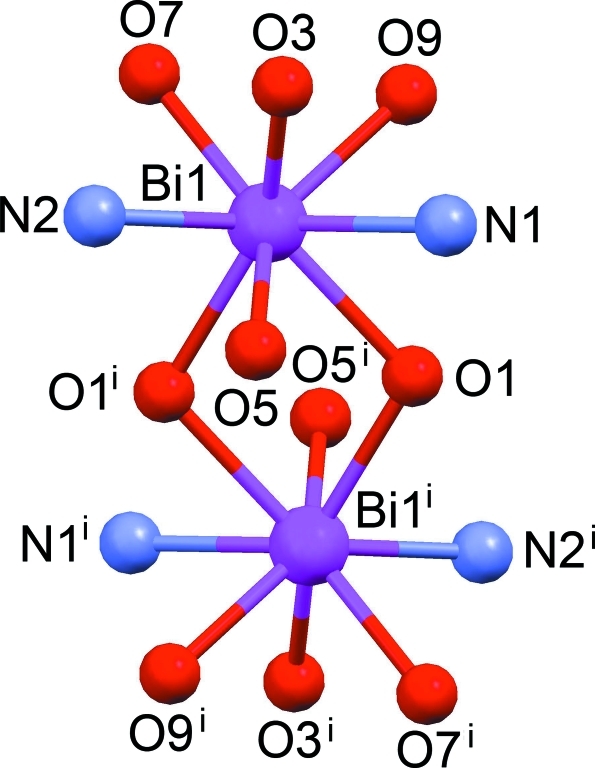
The coordination environment of the BiIII atom, showing a distorted bicapped trigonal-prismatic geometry. [Symmetry code: (i) -x+1, -y, -z+2.]

**Fig. 3.**
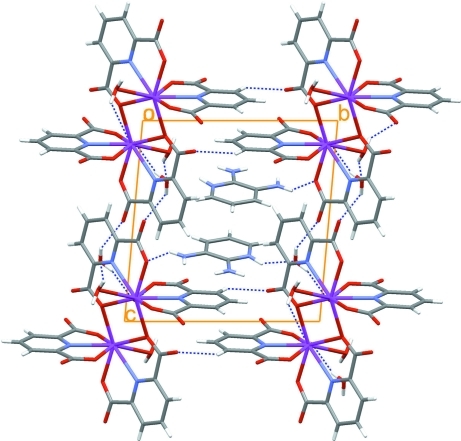
A view of the crystal packing, showing O—H···O, N—H···O and weak C—H···O hydrogen bonds (dashed lines) in the title compound.

**Fig. 4.**
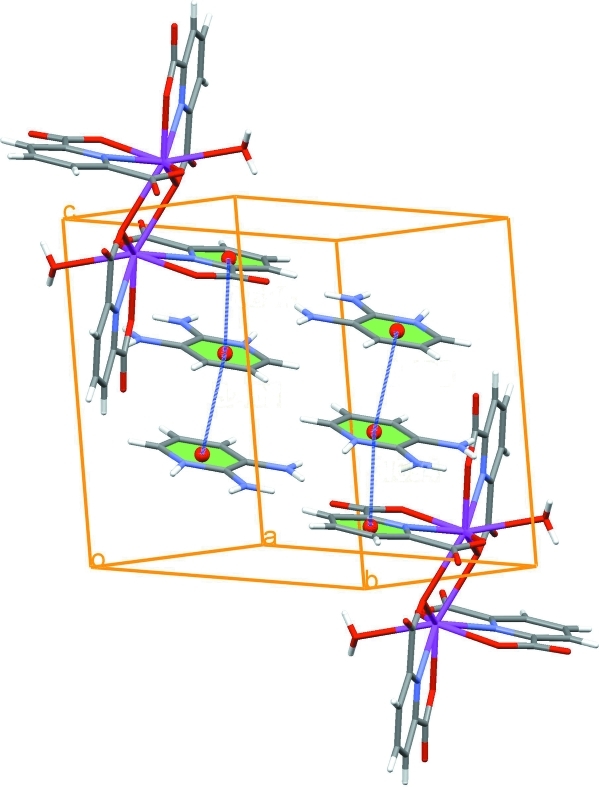
π–π stacking interactions between the aromatic rings of the (2,3-dapyH)+ cation and pydc ligand.

**Fig. 5.**
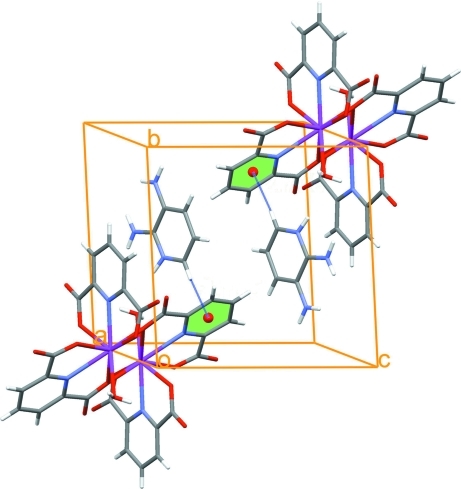
C—H···π interaction between C—H group of the (2,3-dapyH)+ cation and the aromatic ring of the pydc ligand.

## supplementary crystallographic information

### Comment

Pyridine-2,6-dicarboxylic acid (pydcH_2_) can form various complexes containing transition and main metals (Aghabozorg *et al.*, 2008). There are complexes in which pydc acts as a bridging ligand between two metal atoms (Aghabozorg *et al.*, 2010; Sheshmani *et al.*, 2005).

Herein, we report the crystal structure of the title compund as another example of bismuth(III) coordination compound, which bears heterocyclic 2,3-diaminopyridine (2,3-dapy) and pydcH_2_ ligands. The molecular structure of the title compound is shown in Fig. 1. The centrosymmetric binuclear unit consists of two Bi^III^ atoms, four (pydc)^2-^ ligands, two coordinated water molecules. Two pydc ligands act as tridentate ligands with an N atom of the pyridine ring and two O atoms of the dicarboxylate groups acting as donors. One of the dicarboxylate O atoms for the other two pydc ligands plays a bridging role between two Bi atoms. The structure also contains two (2,3-dapyH)^+^ cations and four uncoordinated water molecules. The Bi^III^ atom is eight-coordinated in an N_2_O_6_ environment and has a distorted bicapped trigonal-prismatic geometry, as it is shown in Fig. 2. There are extensive intermolecular O—H···O, N—H···O and weak C—H···O hydrogen bonds, which cause the stability of the crystal structure (Fig. 3, Table 1). There are also π–π interactions between the (2,3-dapyH)^+^ rings and between the (2,3-dapyH)^+^ and pydc rings (Fig. 4), with centroid–centroid distances of 3.489 (5) and 3.694 (5) Å, respectively. Furthermore, there is C—H···π interacton between C—H group of the (2,3-dapyH)^+^ cation and pydc ligand, with an C—H···centroid distance of 2.78 Å (Fig. 5).

### Experimental

An aqueous solution of Bi(NO_3_)_3_ (1 mmol), pydcH_2_ (3 mmol) and 2,3-dapy (1 mmol) was refluxed for about 30 min in a 1:3:1 molar ratio. Brown crystals of the title compound were obtained from the solution by slow evaporation of the solvent within two weeks at room temperature.

### Refinement

H atoms attached to pyridine N and water O atoms were found in a difference Fourier map and refined with *U*_iso_(H) = 1.0–1.5*U*_eq_(N,O). H atoms of the water molecules, H9A, H9B, H10A, H10B, H11A, H11B were refined with distance restraints of O—H = 0.81 (7), 0.84 (8), 0.94 (8), 0.80 (8), 0.89 (8) and 0.92 (9) Å and H···H distance restraints of 1.45 (4) Å for H10A···H10B and 1.40 (4) Å for H11A···H11B. H atoms on C atoms were positioned geometrically and refined as riding atoms, with C—H = 0.93 Å and *U*_iso_(H) = 1.2*U*_eq_(C). The highest residual electron density was found at 0.83 Å from Bi1 atom and the deepest hole at 0.74 Å from Bi1 atom.

### Crystal data

**Table:**

(C_5_H_8_N_3_)_2_[Bi_2_(C_7_H_3_NO_4_)_4_(H_2_O)_2_]·4H_2_O	*Z* = 1
*M**_r_* = 1406.76	*F*(000) = 680
Triclinic, *P*1	*D*_x_ = 2.109 Mg m^−^^3^
Hall symbol: -P 1	Mo *K*α radiation, λ = 0.71073 Å
*a* = 9.3462 (19) Å	Cell parameters from 5940 reflections
*b* = 10.726 (2) Å	θ = 2.2–29.2°
*c* = 11.098 (2) Å	µ = 8.03 mm^−^^1^
α = 95.13 (3)°	*T* = 298 K
β = 91.38 (3)°	Prism, brown
γ = 90.47 (3)°	0.33 × 0.27 × 0.23 mm
*V* = 1107.7 (4) Å^3^	

### Data collection

**Table:**

Stoe IPDS-2 diffractometer	5940 independent reflections
Radiation source: fine-focus sealed tube	5539 reflections with *I* > 2σ(*I*)
graphite	*R*_int_ = 0.113
ω scans	θ_max_ = 29.2°, θ_min_ = 2.2°
Absorption correction: numerical (*X-SHAPE* and *X-RED32*; Stoe & Cie, 2005)	*h* = −12→12
*T*_min_ = 0.083, *T*_max_ = 0.156	*k* = −14→14
12406 measured reflections	*l* = −15→15

### Refinement

**Table:**

Refinement on *F*^2^	Secondary atom site location: difference Fourier map
Least-squares matrix: full	Hydrogen site location: inferred from neighbouring sites
*R*[*F*^2^ > 2σ(*F*^2^)] = 0.057	H atoms treated by a mixture of independent and constrained refinement
*wR*(*F*^2^) = 0.154	*w* = 1/[σ^2^(*F*_o_^2^) + (0.1042*P*)^2^ + 3.0215*P*] where *P* = (*F*_o_^2^ + 2*F*_c_^2^)/3
*S* = 1.05	(Δ/σ)_max_ = 0.001
5940 reflections	Δρ_max_ = 2.98 e Å^−^^3^
347 parameters	Δρ_min_ = −2.93 e Å^−^^3^
9 restraints	Extinction correction: *SHELXL97* (Sheldrick, 2008), Fc^*^=kFc[1+0.001xFc^2^λ^3^/sin(2θ)]^-1/4^
Primary atom site location: structure-invariant direct methods	Extinction coefficient: 0.0047 (13)

### Fractional atomic coordinates and isotropic or equivalent isotropic displacement parameters (Å^2^)

**Table:**

	*x*	*y*	*z*	*U*_iso_*/*U*_eq_	
O10	0.8002 (8)	0.0220 (8)	0.8803 (9)	0.0534 (19)	
O8	−0.0239 (8)	0.3014 (6)	1.0095 (8)	0.056 (2)	
O11	0.9124 (8)	0.8479 (8)	0.7003 (7)	0.0502 (17)	
O6	0.6729 (7)	0.3001 (6)	0.7953 (8)	0.0494 (17)	
Bi1	0.32379 (2)	0.045644 (19)	0.88303 (2)	0.02387 (13)	
N1	0.4091 (7)	−0.0927 (6)	0.7035 (5)	0.0265 (11)	
C5	0.3481 (8)	−0.0744 (7)	0.5968 (7)	0.0297 (14)	
C1	0.5057 (7)	−0.1819 (6)	0.7123 (6)	0.0247 (12)	
C3	0.4867 (12)	−0.2374 (10)	0.4998 (8)	0.047 (2)	
H3	0.5131	−0.2871	0.4312	0.056*	
C7	0.2415 (9)	0.0299 (7)	0.5988 (8)	0.0359 (16)	
C2	0.5469 (10)	−0.2566 (9)	0.6098 (8)	0.0426 (19)	
H2	0.6147	−0.3188	0.6163	0.051*	
C4	0.3855 (10)	−0.1434 (9)	0.4899 (8)	0.0388 (17)	
H4	0.3450	−0.1277	0.4155	0.047*	
N4	1.1655 (8)	0.4867 (7)	0.2534 (8)	0.0436 (18)	
H4A	1.1986	0.4137	0.2319	0.052*	
H4B	1.2133	0.5526	0.2398	0.052*	
C15	1.0421 (8)	0.4974 (7)	0.3068 (7)	0.0300 (14)	
C16	0.9763 (9)	0.6147 (8)	0.3425 (8)	0.0333 (15)	
N3	0.9707 (9)	0.3919 (7)	0.3263 (7)	0.0383 (15)	
N5	1.0463 (9)	0.7230 (6)	0.3244 (9)	0.0462 (19)	
H5A	1.0086	0.7940	0.3469	0.055*	
H5B	1.1281	0.7205	0.2904	0.055*	
C17	0.8460 (10)	0.6133 (9)	0.3960 (10)	0.043 (2)	
H17	0.8021	0.6885	0.4204	0.051*	
C19	0.8402 (11)	0.3898 (9)	0.3806 (9)	0.046 (2)	
H19	0.7959	0.3144	0.3938	0.055*	
C18	0.7782 (10)	0.5004 (11)	0.4144 (10)	0.048 (2)	
H18	0.6892	0.5012	0.4502	0.057*	
O3	0.2133 (7)	0.0852 (6)	0.7009 (6)	0.0381 (13)	
O7	0.0896 (6)	0.1326 (5)	0.9346 (6)	0.0340 (12)	
O5	0.5370 (6)	0.1332 (5)	0.8106 (6)	0.0324 (11)	
N2	0.3143 (7)	0.2700 (6)	0.8827 (6)	0.0262 (11)	
C8	0.4311 (8)	0.3335 (7)	0.8530 (7)	0.0283 (13)	
O4	0.1860 (11)	0.0529 (9)	0.5024 (7)	0.066 (2)	
C14	0.0788 (9)	0.2486 (7)	0.9591 (8)	0.0331 (15)	
C13	0.5562 (8)	0.2518 (7)	0.8158 (7)	0.0290 (14)	
C12	0.1996 (8)	0.3318 (7)	0.9215 (7)	0.0294 (14)	
C9	0.4360 (8)	0.4623 (7)	0.8582 (8)	0.0321 (15)	
H9	0.5173	0.5045	0.8362	0.039*	
C11	0.1952 (8)	0.4614 (7)	0.9293 (8)	0.0324 (15)	
H11	0.1135	0.5036	0.9554	0.039*	
C10	0.3145 (9)	0.5268 (7)	0.8975 (9)	0.0358 (17)	
H10	0.3137	0.6138	0.9023	0.043*	
O1	0.5240 (6)	−0.1236 (5)	0.9223 (5)	0.0290 (10)	
O2	0.6605 (9)	−0.2791 (7)	0.8471 (6)	0.0489 (17)	
C6	0.5695 (8)	−0.1989 (7)	0.8366 (7)	0.0276 (13)	
O9	0.1606 (8)	−0.1496 (7)	0.8422 (7)	0.0455 (15)	
H10B	0.867 (9)	0.064 (12)	0.906 (13)	0.055*	
H11A	0.851 (13)	0.845 (14)	0.638 (9)	0.068*	
H11B	0.860 (15)	0.908 (13)	0.742 (11)	0.068*	
H9A	0.143 (14)	−0.171 (15)	0.909 (8)	0.068*	
H9B	0.090 (13)	−0.126 (14)	0.802 (10)	0.068*	
H10A	0.717 (9)	0.070 (11)	0.875 (12)	0.055*	
H3A	1.007 (14)	0.325 (13)	0.289 (12)	0.055*	

### Atomic displacement parameters (Å^2^)

**Table:**

	*U*^11^	*U*^22^	*U*^33^	*U*^12^	*U*^13^	*U*^23^
O10	0.036 (3)	0.043 (3)	0.081 (6)	0.007 (3)	−0.001 (3)	0.005 (4)
O8	0.052 (4)	0.029 (3)	0.090 (6)	0.012 (3)	0.045 (4)	0.005 (3)
O11	0.049 (4)	0.054 (4)	0.049 (4)	0.022 (3)	−0.006 (3)	0.011 (3)
O6	0.034 (3)	0.034 (3)	0.080 (5)	−0.004 (2)	0.025 (3)	0.001 (3)
Bi1	0.02420 (17)	0.01863 (16)	0.02853 (18)	0.00371 (9)	0.00376 (9)	−0.00045 (9)
N1	0.030 (3)	0.026 (3)	0.024 (3)	0.004 (2)	0.004 (2)	0.000 (2)
C5	0.032 (3)	0.025 (3)	0.032 (4)	0.000 (3)	0.003 (3)	0.000 (3)
C1	0.025 (3)	0.022 (3)	0.026 (3)	0.002 (2)	0.003 (2)	−0.004 (2)
C3	0.060 (6)	0.049 (5)	0.030 (4)	0.014 (4)	0.012 (4)	−0.010 (4)
C7	0.039 (4)	0.028 (3)	0.042 (4)	0.006 (3)	0.004 (3)	0.006 (3)
C2	0.041 (4)	0.046 (5)	0.037 (4)	0.011 (4)	0.006 (3)	−0.015 (4)
C4	0.045 (4)	0.043 (4)	0.027 (4)	0.004 (3)	0.006 (3)	−0.003 (3)
N4	0.037 (4)	0.030 (3)	0.063 (5)	0.005 (3)	0.019 (3)	−0.004 (3)
C15	0.035 (4)	0.030 (3)	0.025 (3)	0.000 (3)	−0.001 (3)	0.002 (3)
C16	0.032 (4)	0.029 (3)	0.039 (4)	0.000 (3)	0.005 (3)	0.003 (3)
N3	0.045 (4)	0.027 (3)	0.043 (4)	−0.001 (3)	0.010 (3)	−0.002 (3)
N5	0.051 (4)	0.019 (3)	0.069 (5)	0.007 (3)	0.024 (4)	0.002 (3)
C17	0.037 (4)	0.036 (4)	0.055 (6)	0.012 (3)	0.013 (4)	0.002 (4)
C19	0.053 (5)	0.041 (4)	0.044 (5)	−0.009 (4)	0.013 (4)	0.003 (4)
C18	0.033 (4)	0.057 (6)	0.052 (5)	−0.011 (4)	0.015 (4)	−0.008 (4)
O3	0.044 (3)	0.034 (3)	0.036 (3)	0.018 (2)	−0.007 (2)	0.001 (2)
O7	0.029 (2)	0.024 (2)	0.048 (3)	−0.0006 (19)	0.007 (2)	0.004 (2)
O5	0.031 (3)	0.024 (2)	0.042 (3)	0.0096 (19)	0.014 (2)	0.002 (2)
N2	0.027 (3)	0.025 (3)	0.026 (3)	0.005 (2)	0.006 (2)	−0.002 (2)
C8	0.033 (3)	0.022 (3)	0.029 (3)	0.002 (2)	0.006 (3)	0.001 (2)
O4	0.082 (6)	0.073 (5)	0.042 (4)	0.035 (5)	−0.015 (4)	0.003 (4)
C14	0.033 (4)	0.024 (3)	0.043 (4)	0.005 (3)	0.008 (3)	0.008 (3)
C13	0.027 (3)	0.027 (3)	0.032 (3)	0.004 (2)	0.008 (3)	−0.001 (3)
C12	0.028 (3)	0.025 (3)	0.035 (4)	0.002 (2)	0.005 (3)	−0.003 (3)
C9	0.026 (3)	0.025 (3)	0.046 (4)	0.003 (2)	0.008 (3)	0.003 (3)
C11	0.028 (3)	0.026 (3)	0.043 (4)	0.006 (3)	0.007 (3)	−0.001 (3)
C10	0.036 (4)	0.023 (3)	0.048 (5)	0.007 (3)	0.006 (3)	0.000 (3)
O1	0.030 (2)	0.032 (2)	0.023 (2)	0.007 (2)	−0.0015 (19)	−0.0062 (19)
O2	0.067 (4)	0.040 (3)	0.039 (3)	0.029 (3)	−0.002 (3)	−0.002 (3)
C6	0.027 (3)	0.026 (3)	0.029 (3)	0.004 (2)	0.006 (3)	−0.002 (3)
O9	0.045 (3)	0.041 (3)	0.049 (4)	−0.012 (3)	0.010 (3)	−0.005 (3)

### Geometric parameters (Å, °)

**Table:**

O10—H10B	0.80 (8)	C15—C16	1.433 (11)
O10—H10A	0.94 (8)	C16—N5	1.362 (11)
O8—C14	1.239 (10)	C16—C17	1.368 (12)
O11—H11A	0.89 (8)	N3—C19	1.374 (12)
O11—H11B	0.92 (9)	N3—H3A	0.87 (14)
O6—C13	1.238 (9)	N5—H5A	0.8600
Bi1—O3	2.323 (6)	N5—H5B	0.8600
Bi1—O5	2.384 (6)	C17—C18	1.396 (15)
Bi1—N2	2.409 (6)	C17—H17	0.9300
Bi1—O7	2.447 (6)	C19—C18	1.351 (15)
Bi1—N1	2.525 (6)	C19—H19	0.9300
Bi1—O9	2.581 (7)	C18—H18	0.9300
Bi1—O1^i^	2.627 (5)	O7—C14	1.255 (9)
Bi1—O1	2.673 (5)	O5—C13	1.279 (9)
N1—C1	1.329 (9)	N2—C12	1.325 (9)
N1—C5	1.333 (10)	N2—C8	1.346 (9)
C5—C4	1.394 (11)	C8—C9	1.377 (10)
C5—C7	1.504 (11)	C8—C13	1.510 (10)
C1—C2	1.395 (10)	C14—C12	1.522 (11)
C1—C6	1.516 (10)	C12—C11	1.386 (10)
C3—C2	1.364 (14)	C9—C10	1.394 (10)
C3—C4	1.398 (14)	C9—H9	0.9300
C3—H3	0.9300	C11—C10	1.381 (12)
C7—O4	1.224 (12)	C11—H11	0.9300
C7—O3	1.266 (11)	C10—H10	0.9300
C2—H2	0.9300	O1—C6	1.275 (9)
C4—H4	0.9300	O1—Bi1^i^	2.627 (5)
N4—C15	1.310 (11)	O2—C6	1.225 (10)
N4—H4A	0.8600	O9—H9A	0.81 (7)
N4—H4B	0.8600	O9—H9B	0.84 (8)
C15—N3	1.346 (10)		
			
H10B—O10—H10A	112 (9)	N3—C15—C16	117.8 (7)
H11A—O11—H11B	91 (8)	N5—C16—C17	122.4 (8)
O3—Bi1—O5	87.5 (2)	N5—C16—C15	119.2 (7)
O3—Bi1—N2	73.7 (2)	C17—C16—C15	118.4 (8)
O5—Bi1—N2	67.26 (19)	C15—N3—C19	124.1 (8)
O3—Bi1—O7	74.0 (2)	C15—N3—H3A	113 (9)
O5—Bi1—O7	133.22 (18)	C19—N3—H3A	122 (9)
N2—Bi1—O7	66.40 (19)	C16—N5—H5A	120.0
O3—Bi1—N1	66.3 (2)	C16—N5—H5B	120.0
O5—Bi1—N1	70.7 (2)	H5A—N5—H5B	120.0
N2—Bi1—N1	122.1 (2)	C16—C17—C18	120.9 (8)
O7—Bi1—N1	132.5 (2)	C16—C17—H17	119.6
O3—Bi1—O9	79.0 (3)	C18—C17—H17	119.6
O5—Bi1—O9	140.1 (2)	C18—C19—N3	118.1 (9)
N2—Bi1—O9	140.2 (2)	C18—C19—H19	121.0
O7—Bi1—O9	78.7 (2)	N3—C19—H19	121.0
N1—Bi1—O9	69.4 (2)	C19—C18—C17	120.7 (8)
O3—Bi1—O1^i^	150.1 (2)	C19—C18—H18	119.6
O5—Bi1—O1^i^	74.6 (2)	C17—C18—H18	119.6
N2—Bi1—O1^i^	77.3 (2)	C7—O3—Bi1	124.9 (5)
O7—Bi1—O1^i^	100.97 (19)	C14—O7—Bi1	119.0 (5)
N1—Bi1—O1^i^	126.40 (18)	C13—O5—Bi1	121.0 (4)
O9—Bi1—O1^i^	129.7 (2)	C12—N2—C8	119.8 (6)
O3—Bi1—O1	128.33 (18)	C12—N2—Bi1	120.5 (5)
O5—Bi1—O1	76.04 (18)	C8—N2—Bi1	119.4 (5)
N2—Bi1—O1	136.45 (19)	N2—C8—C9	122.5 (7)
O7—Bi1—O1	147.79 (19)	N2—C8—C13	114.3 (6)
N1—Bi1—O1	61.99 (18)	C9—C8—C13	123.2 (7)
O9—Bi1—O1	83.3 (2)	O8—C14—O7	125.2 (8)
O1^i^—Bi1—O1	70.87 (18)	O8—C14—C12	117.1 (7)
C1—N1—C5	120.5 (6)	O7—C14—C12	117.7 (7)
C1—N1—Bi1	123.4 (5)	O6—C13—O5	122.5 (7)
C5—N1—Bi1	116.0 (5)	O6—C13—C8	120.1 (7)
N1—C5—C4	122.3 (7)	O5—C13—C8	117.4 (6)
N1—C5—C7	115.4 (7)	N2—C12—C11	121.4 (7)
C4—C5—C7	122.3 (8)	N2—C12—C14	114.3 (6)
N1—C1—C2	120.7 (7)	C11—C12—C14	124.3 (7)
N1—C1—C6	117.8 (6)	C8—C9—C10	117.5 (7)
C2—C1—C6	121.5 (7)	C8—C9—H9	121.3
C2—C3—C4	120.3 (8)	C10—C9—H9	121.3
C2—C3—H3	119.8	C10—C11—C12	118.8 (7)
C4—C3—H3	119.8	C10—C11—H11	120.6
O4—C7—O3	124.9 (8)	C12—C11—H11	120.6
O4—C7—C5	117.8 (8)	C11—C10—C9	119.9 (7)
O3—C7—C5	117.3 (7)	C11—C10—H10	120.0
C3—C2—C1	119.3 (8)	C9—C10—H10	120.0
C3—C2—H2	120.3	C6—O1—Bi1^i^	124.2 (5)
C1—C2—H2	120.3	C6—O1—Bi1	121.5 (5)
C5—C4—C3	116.9 (8)	Bi1^i^—O1—Bi1	109.13 (18)
C5—C4—H4	121.6	O2—C6—O1	125.5 (7)
C3—C4—H4	121.6	O2—C6—C1	119.2 (7)
C15—N4—H4A	120.0	O1—C6—C1	115.2 (6)
C15—N4—H4B	120.0	Bi1—O9—H9A	105 (10)
H4A—N4—H4B	120.0	Bi1—O9—H9B	106 (10)
N4—C15—N3	118.1 (8)	H9A—O9—H9B	116 (10)
N4—C15—C16	124.0 (7)		
			
O3—Bi1—N1—C1	−178.5 (6)	O1^i^—Bi1—O5—C13	−75.3 (6)
O5—Bi1—N1—C1	85.6 (6)	O1—Bi1—O5—C13	−148.9 (6)
N2—Bi1—N1—C1	131.0 (5)	O3—Bi1—N2—C12	86.6 (6)
O7—Bi1—N1—C1	−142.8 (5)	O5—Bi1—N2—C12	−179.2 (6)
O9—Bi1—N1—C1	−91.8 (6)	O7—Bi1—N2—C12	7.3 (6)
O1^i^—Bi1—N1—C1	33.0 (6)	N1—Bi1—N2—C12	134.0 (6)
O1—Bi1—N1—C1	1.6 (5)	O9—Bi1—N2—C12	38.0 (7)
O3—Bi1—N1—C5	−0.5 (5)	O1^i^—Bi1—N2—C12	−100.8 (6)
O5—Bi1—N1—C5	−96.4 (5)	O1—Bi1—N2—C12	−144.4 (5)
N2—Bi1—N1—C5	−51.0 (6)	O3—Bi1—N2—C8	−99.3 (6)
O7—Bi1—N1—C5	35.2 (6)	O5—Bi1—N2—C8	−5.1 (5)
O9—Bi1—N1—C5	86.2 (5)	O7—Bi1—N2—C8	−178.5 (6)
O1^i^—Bi1—N1—C5	−149.0 (5)	N1—Bi1—N2—C8	−51.9 (6)
O1—Bi1—N1—C5	179.6 (6)	O9—Bi1—N2—C8	−147.8 (5)
C1—N1—C5—C4	−2.4 (11)	O1^i^—Bi1—N2—C8	73.3 (5)
Bi1—N1—C5—C4	179.5 (6)	O1—Bi1—N2—C8	29.8 (7)
C1—N1—C5—C7	−179.6 (7)	C12—N2—C8—C9	−1.7 (12)
Bi1—N1—C5—C7	2.4 (8)	Bi1—N2—C8—C9	−175.9 (6)
C5—N1—C1—C2	1.1 (11)	C12—N2—C8—C13	177.3 (7)
Bi1—N1—C1—C2	179.0 (6)	Bi1—N2—C8—C13	3.1 (9)
C5—N1—C1—C6	−179.2 (6)	Bi1—O7—C14—O8	−164.6 (8)
Bi1—N1—C1—C6	−1.3 (9)	Bi1—O7—C14—C12	16.9 (10)
N1—C5—C7—O4	177.7 (9)	Bi1—O5—C13—O6	169.5 (7)
C4—C5—C7—O4	0.5 (13)	Bi1—O5—C13—C8	−8.4 (10)
N1—C5—C7—O3	−4.0 (11)	N2—C8—C13—O6	−174.6 (8)
C4—C5—C7—O3	178.8 (8)	C9—C8—C13—O6	4.4 (13)
C4—C3—C2—C1	0.4 (16)	N2—C8—C13—O5	3.3 (11)
N1—C1—C2—C3	−0.2 (14)	C9—C8—C13—O5	−177.7 (8)
C6—C1—C2—C3	−179.8 (9)	C8—N2—C12—C11	1.8 (12)
N1—C5—C4—C3	2.6 (13)	Bi1—N2—C12—C11	175.9 (6)
C7—C5—C4—C3	179.5 (9)	C8—N2—C12—C14	−176.5 (7)
C2—C3—C4—C5	−1.5 (15)	Bi1—N2—C12—C14	−2.4 (9)
N4—C15—C16—N5	−2.3 (13)	O8—C14—C12—N2	171.6 (9)
N3—C15—C16—N5	179.7 (9)	O7—C14—C12—N2	−9.8 (11)
N4—C15—C16—C17	179.0 (9)	O8—C14—C12—C11	−6.6 (14)
N3—C15—C16—C17	1.0 (12)	O7—C14—C12—C11	172.0 (8)
N4—C15—N3—C19	−179.6 (9)	N2—C8—C9—C10	0.8 (13)
C16—C15—N3—C19	−1.5 (13)	C13—C8—C9—C10	−178.1 (8)
N5—C16—C17—C18	−179.2 (11)	N2—C12—C11—C10	−1.0 (13)
C15—C16—C17—C18	−0.6 (15)	C14—C12—C11—C10	177.1 (8)
C15—N3—C19—C18	1.4 (15)	C12—C11—C10—C9	0.2 (13)
N3—C19—C18—C17	−0.9 (17)	C8—C9—C10—C11	−0.1 (13)
C16—C17—C18—C19	0.6 (17)	O3—Bi1—O1—C6	−2.0 (7)
O4—C7—O3—Bi1	−178.0 (9)	O5—Bi1—O1—C6	−77.3 (6)
C5—C7—O3—Bi1	3.8 (11)	N2—Bi1—O1—C6	−110.2 (6)
O5—Bi1—O3—C7	68.1 (7)	O7—Bi1—O1—C6	124.4 (6)
N2—Bi1—O3—C7	135.2 (8)	N1—Bi1—O1—C6	−1.9 (5)
O7—Bi1—O3—C7	−155.3 (8)	O9—Bi1—O1—C6	68.3 (6)
N1—Bi1—O3—C7	−1.9 (7)	O1^i^—Bi1—O1—C6	−155.5 (7)
O9—Bi1—O3—C7	−74.1 (7)	O3—Bi1—O1—Bi1^i^	153.5 (2)
O1^i^—Bi1—O3—C7	120.6 (7)	O5—Bi1—O1—Bi1^i^	78.3 (2)
O1—Bi1—O3—C7	−1.8 (8)	N2—Bi1—O1—Bi1^i^	45.4 (4)
O3—Bi1—O7—C14	−91.9 (7)	O7—Bi1—O1—Bi1^i^	−80.0 (4)
O5—Bi1—O7—C14	−21.3 (8)	N1—Bi1—O1—Bi1^i^	153.6 (3)
N2—Bi1—O7—C14	−13.0 (6)	O9—Bi1—O1—Bi1^i^	−136.2 (3)
N1—Bi1—O7—C14	−125.7 (6)	O1^i^—Bi1—O1—Bi1^i^	0.0
O9—Bi1—O7—C14	−173.6 (7)	Bi1^i^—O1—C6—O2	28.4 (11)
O1^i^—Bi1—O7—C14	57.8 (7)	Bi1—O1—C6—O2	−179.9 (7)
O1—Bi1—O7—C14	129.2 (6)	Bi1^i^—O1—C6—C1	−149.8 (5)
O3—Bi1—O5—C13	80.5 (6)	Bi1—O1—C6—C1	2.0 (8)
N2—Bi1—O5—C13	7.2 (6)	N1—C1—C6—O2	−178.8 (8)
O7—Bi1—O5—C13	15.4 (7)	C2—C1—C6—O2	0.9 (12)
N1—Bi1—O5—C13	146.3 (7)	N1—C1—C6—O1	−0.6 (10)
O9—Bi1—O5—C13	150.0 (6)	C2—C1—C6—O1	179.1 (8)

Symmetry codes: (i) −*x*+1, −*y*, −*z*+2.

### Hydrogen-bond geometry (Å, °)

**Table:**

*D*—H···*A*	*D*—H	H···*A*	*D*···*A*	*D*—H···*A*
N3—H3A···O11^ii^	0.87 (14)	2.02 (14)	2.797 (10)	148 (12)
N4—H4A···O2^iii^	0.86	2.11	2.924 (9)	158
N4—H4B···O6^ii^	0.86	1.97	2.830 (10)	178
N5—H5A···O3^iv^	0.86	2.53	3.216 (10)	138
N5—H5B···O6^ii^	0.86	2.11	2.972 (10)	176
O9—H9A···O8^v^	0.81 (7)	2.07 (12)	2.746 (10)	141 (15)
O9—H9B···O11^vi^	0.84 (8)	1.99 (10)	2.771 (11)	155 (15)
O10—H10A···O5	0.94 (8)	1.96 (8)	2.862 (9)	160 (11)
O10—H10B···O7^vii^	0.80 (8)	2.21 (8)	2.972 (10)	160 (13)
O11—H11A···O4^iv^	0.89 (8)	2.01 (13)	2.717 (11)	136 (14)
O11—H11B···O10^viii^	0.92 (9)	1.96 (9)	2.836 (12)	158 (13)
C11—H11···O8^ix^	0.93	2.26	3.050 (10)	142

Symmetry codes: (ii) −*x*+2, −*y*+1, −*z*+1; (iii) −*x*+2, −*y*, −*z*+1; (iv) −*x*+1, −*y*+1, −*z*+1; (v) −*x*, −*y*, −*z*+2; (vi) *x*−1, *y*−1, *z*; (vii) *x*+1, *y*, *z*; (viii) *x*, *y*+1, *z*; (ix) −*x*, −*y*+1, −*z*+2.
